# 2-(3-Meth­oxy­phen­oxy)benzoic acid

**DOI:** 10.1107/S1600536811011019

**Published:** 2011-04-07

**Authors:** Zhi-Fang Zhang

**Affiliations:** a School of Chemistry and Chemical Engineering, Yulin University, Yulin 719000, People’s Republic of China

## Abstract

In the crystal structure of the title compound, C_14_H_12_O_4_, the mol­ecules form classical O—H⋯O hydrogen-bonded carb­oxy­lic acid dimers. These dimers are linked by C—H⋯pi; inter­actions into a three-dimensional network. The benzene rings are oriented at a dihedral angle of 69.6 (3)°.

## Related literature

For applications of the title compound, see: Jackson *et al.* (1993) ▶; Gapinski *et al.* (1990 ▶). For related structures, see: Shi *et al.* (2011 ▶); Raghunathan *et al.* (1982 ▶). For the synthesis of the title compound, see: Pellón *et al.* (1995 ▶). For bond-length data, see: Allen *et al.* (1987 ▶).
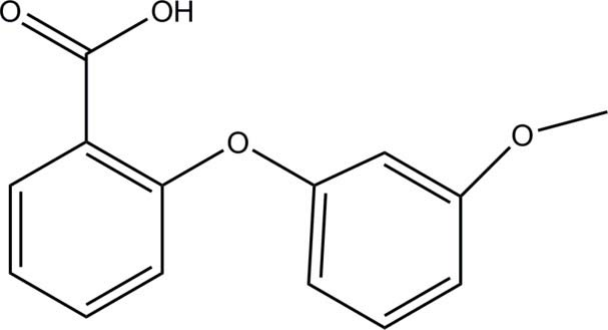


## Experimental

### 

#### Crystal data

C_14_H_12_O_4_
*M* *_r_* = 244.24
Orthorhombic, 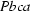

*a* = 14.309 (3) Å
*b* = 8.5330 (17) Å
*c* = 19.432 (4) Å
*V* = 2372.6 (8) Å^3^
*Z* = 8
Mo *K*α radiation
μ = 0.10 mm^−1^
*T* = 295 K
0.30 × 0.10 × 0.05 mm

#### Data collection

Enraf–Nonius CAD-4 diffractometer
Absorption correction: ψ scan (North *et al.*, 1968 ▶) *T*_min_ = 0.970, *T*_max_ = 0.995
4273 measured reflections
2175 independent reflections
1056 reflections with *I* > 2σ(*I*)
*R*_int_ = 0.093
3 standard reflections every 200 reflections  intensity decay: 1%

#### Refinement

*R*[*F* ^2^ > 2σ(*F* ^2^)] = 0.062
*wR*(*F* ^2^) = 0.133
*S* = 1.00
2175 reflections
163 parameters
H-atom parameters constrained
Δρ_max_ = 0.16 e Å^−3^
Δρ_min_ = −0.16 e Å^−3^

### 

Data collection: *CAD-4 Software* (Enraf–Nonius, 1985 ▶); cell refinement: *CAD-4 Software*; data reduction: *XCAD4* (Harms & Wocadlo, 1995 ▶); program(s) used to solve structure: *SHELXS97* (Sheldrick, 2008 ▶); program(s) used to refine structure: *SHELXL97* (Sheldrick, 2008 ▶); molecular graphics: *SHELXTL* (Sheldrick, 2008 ▶); software used to prepare material for publication: *SHELXTL*.

## Supplementary Material

Crystal structure: contains datablocks I, global. DOI: 10.1107/S1600536811011019/hg5013sup1.cif

Structure factors: contains datablocks I. DOI: 10.1107/S1600536811011019/hg5013Isup2.hkl

Additional supplementary materials:  crystallographic information; 3D view; checkCIF report

## Figures and Tables

**Table 1:** Hydrogen-bond geometry (Å, °) *Cg*1 is the centroid of the C2–C7 ring.

*D*—H⋯*A*	*D*—H	H⋯*A*	*D*⋯*A*	*D*—H⋯*A*
O4—H4*A*⋯O3^i^	0.82	1.82	2.633 (3)	173
C1—H1*B*⋯*Cg*1^ii^	0.96	2.89	3.784 (4)	155

Symmetry codes: (i) 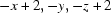; (ii) 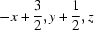.

**Fig. 1.**
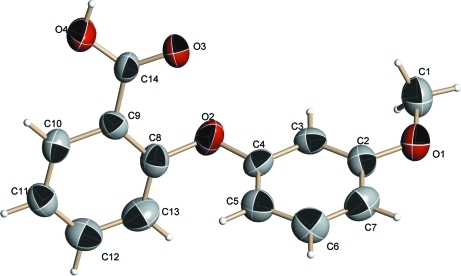
The molecular structure of (I) (thermal ellipsoids are shown at 30% probability levels).

**Fig. 2.**
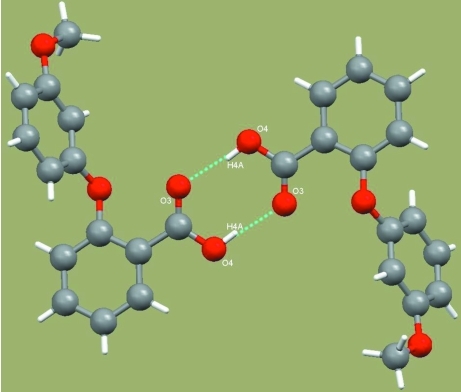
The structure of a dimer of (I).

## supplementary crystallographic information

### Comment

The title compound, 2-(3-methoxyphenoxy)benzoic acid is an important intermediate of xanthone dicarboxylic acids (Jackson *et al.*, 1972). Xanthone dicarboxylic acid, such as LY223982, which inhibited the binding of LTB4 to receptors on intact human neutrophils nearly as well as nonradioactive LTB4 (Gapinski *et al.*, 1990). Knowledge of the crystal structure of such benzoicacid derivatives gives us not only information about nuclearity of the complex molecule, but is important in understanding the behaviour of these compounds with respect to the mechanisms of pharmacological activities and physiological activities. Therefore, we have synthesized the title compound, (I), and report its crystal structure here.

The molecular structure of (I) is shown in Fig. 1, and the intermolecular O—H···O hydrogen bond (Table 1) results in the formation of carboxylic acid dimers (Fig. 2.). The bond lengths are within normal ranges (Allen *et al.*, 1987). Similar crystal structure of some compounds have been reported (Shi *et al.*, 2011; Raghunathan *et al.*, 1982).

In the molecule of (I), the dihedral angle of the rings(C3—C6) and (C8—C13) is 69.6 (3)°, the molecules were connected together *via* O—H···O intermolecular hydrogen bonds to form dimers. These dimers are linked by C—H···π and weak C—H···O interactions to give a three-dimensional network, which seems to be very effective in the stabilization of the crystal structure.

### Experimental

The title compound (I) was prepared by the method of Ullmann condensation reaction reported in literature (Pellón *et al.*, 1995). A mixture of 2-chlorobenzoic acid (6.26 g, 0.04 mol), 3-methoxyphenol (9.93 g, 0.08 mol), anhydrous K_2_CO_3_ (11.04 g' 0.08 mol), pyridine (1.58 g' 0.02 mol), Cu powder (0.2 g) and cuprous iodide (0.2 g) in 25 ml water was kept at reflux for two hours. The mixture was then basified with Na_2_CO_3_ solution and extracted with diethyl ether. The aqueous solution was acidified with HCl, the precipitated solid was filtered off and disolved in NaOH; the basic solution was filtered (charcoal) and acidified with acetic acid. The 2-(3-methoxyphenoxy)benzoic acid was crystalized from the mixture.

### Refinement

H atoms were positioned geometrically and refined as riding groups, with O—H = 0.82 and C—H = 0.93 Å for aromatic H, and constrained to ride on their parent atoms, with *U*_iso_(H) = *xU*_eq_(C), where *x* = 1.2 for aromatic H, and *x* = 1.5 for other H.

### Crystal data

**Table:**

C_14_H_12_O_4_	*F*(000) = 1024
*M**_r_* = 244.24	*D*_x_ = 1.367 Mg m^−^^3^
Orthorhombic, *P**b**c**a*	Mo *K*α radiation, λ = 0.71073 Å
Hall symbol: -P 2ac 2ab	Cell parameters from 25 reflections
*a* = 14.309 (3) Å	θ = 9–12°
*b* = 8.5330 (17) Å	µ = 0.10 mm^−^^1^
*c* = 19.432 (4) Å	*T* = 295 K
*V* = 2372.6 (8) Å^3^	Block, colourless
*Z* = 8	0.30 × 0.10 × 0.05 mm

### Data collection

**Table:**

Enraf–Nonius CAD-4 diffractometer	1056 reflections with *I* > 2σ(*I*)
Radiation source: fine-focus sealed tube	*R*_int_ = 0.093
graphite	θ_max_ = 25.4°, θ_min_ = 2.1°
ω/2θ scans	*h* = 0→17
Absorption correction: ψ scan (North *et al.*, 1968)	*k* = 0→10
*T*_min_ = 0.970, *T*_max_ = 0.995	*l* = −23→23
4273 measured reflections	3 standard reflections every 200 reflections
2175 independent reflections	intensity decay: 1%

### Refinement

**Table:**

Refinement on *F*^2^	Primary atom site location: structure-invariant direct methods
Least-squares matrix: full	Secondary atom site location: difference Fourier map
*R*[*F*^2^ > 2σ(*F*^2^)] = 0.062	Hydrogen site location: inferred from neighbouring sites
*wR*(*F*^2^) = 0.133	H-atom parameters constrained
*S* = 1.00	*w* = 1/[σ^2^(*F*_o_^2^) + (0.039*P*)^2^] where *P* = (*F*_o_^2^ + 2*F*_c_^2^)/3
2175 reflections	(Δ/σ)_max_ < 0.001
163 parameters	Δρ_max_ = 0.16 e Å^−^^3^
0 restraints	Δρ_min_ = −0.16 e Å^−^^3^

### Special details

**Table:**

Geometry. All e.s.d.'s (except the e.s.d. in the dihedral angle between two l.s. planes) are estimated using the full covariance matrix. The cell e.s.d.'s are taken into account individually in the estimation of e.s.d.'s in distances, angles and torsion angles; correlations between e.s.d.'s in cell parameters are only used when they are defined by crystal symmetry. An approximate (isotropic) treatment of cell e.s.d.'s is used for estimating e.s.d.'s involving l.s. planes.
Refinement. Refinement of *F*^2^ against ALL reflections. The weighted *R*-factor *wR* and goodness of fit *S* are based on *F*^2^, conventional *R*-factors *R* are based on *F*, with *F* set to zero for negative *F*^2^. The threshold expression of *F*^2^ > σ(*F*^2^) is used only for calculating *R*-factors(gt) *etc*. and is not relevant to the choice of reflections for refinement. *R*-factors based on *F*^2^ are statistically about twice as large as those based on *F*, and *R*- factors based on ALL data will be even larger.

### Fractional atomic coordinates and isotropic or equivalent isotropic displacement parameters (Å^2^)

**Table:**

	*x*	*y*	*z*	*U*_iso_*/*U*_eq_	
O1	0.60412 (16)	0.2617 (3)	0.77635 (10)	0.0641 (7)	
C1	0.6361 (3)	0.4135 (4)	0.79576 (18)	0.0738 (11)	
H1A	0.6239	0.4863	0.7592	0.111*	
H1B	0.7021	0.4098	0.8046	0.111*	
H1C	0.6040	0.4468	0.8366	0.111*	
O2	0.70190 (16)	0.0652 (2)	0.99264 (12)	0.0668 (7)	
C2	0.6162 (2)	0.1419 (4)	0.82240 (16)	0.0510 (8)	
O3	0.88741 (15)	0.0003 (3)	0.97959 (10)	0.0654 (7)	
C3	0.6528 (2)	0.1601 (4)	0.88657 (16)	0.0495 (8)	
H3A	0.6720	0.2584	0.9016	0.059*	
O4	0.95145 (16)	−0.0699 (3)	1.07827 (11)	0.0783 (8)	
H4A	0.9992	−0.0465	1.0573	0.117*	
C4	0.6614 (2)	0.0306 (4)	0.92945 (16)	0.0472 (8)	
C5	0.6323 (2)	−0.1143 (4)	0.90941 (18)	0.0570 (9)	
H5A	0.6371	−0.2000	0.9388	0.068*	
C6	0.5953 (3)	−0.1298 (4)	0.84420 (18)	0.0660 (10)	
H6A	0.5751	−0.2279	0.8296	0.079*	
C7	0.5878 (2)	−0.0056 (5)	0.80078 (18)	0.0638 (10)	
H7A	0.5636	−0.0193	0.7568	0.077*	
C8	0.7034 (2)	−0.0465 (4)	1.04428 (17)	0.0533 (8)	
C9	0.7889 (2)	−0.0922 (3)	1.06981 (15)	0.0454 (7)	
C10	0.7896 (2)	−0.1884 (4)	1.12863 (16)	0.0535 (9)	
H10A	0.8463	−0.2195	1.1477	0.064*	
C11	0.7077 (3)	−0.2363 (4)	1.15792 (17)	0.0598 (9)	
H11A	0.7090	−0.3012	1.1964	0.072*	
C12	0.6238 (3)	−0.1898 (5)	1.1312 (2)	0.0666 (10)	
H12A	0.5683	−0.2236	1.1512	0.080*	
C13	0.6218 (2)	−0.0919 (5)	1.0743 (2)	0.0699 (10)	
H13A	0.5650	−0.0575	1.0567	0.084*	
C14	0.8796 (2)	−0.0495 (4)	1.03863 (15)	0.0488 (8)	

### Atomic displacement parameters (Å^2^)

**Table:**

	*U*^11^	*U*^22^	*U*^33^	*U*^12^	*U*^13^	*U*^23^
O1	0.0741 (17)	0.0676 (16)	0.0506 (13)	0.0054 (14)	−0.0066 (12)	0.0027 (13)
C1	0.091 (3)	0.067 (3)	0.064 (2)	0.008 (2)	−0.008 (2)	0.010 (2)
O2	0.0799 (17)	0.0490 (13)	0.0716 (15)	−0.0051 (12)	−0.0318 (14)	0.0080 (12)
C2	0.049 (2)	0.055 (2)	0.0488 (19)	0.0094 (18)	0.0042 (15)	0.0037 (18)
O3	0.0670 (15)	0.0839 (17)	0.0452 (12)	−0.0097 (13)	−0.0051 (11)	0.0177 (14)
C3	0.046 (2)	0.0420 (19)	0.061 (2)	−0.0060 (16)	−0.0011 (16)	−0.0044 (16)
O4	0.0547 (15)	0.115 (2)	0.0654 (16)	−0.0057 (16)	−0.0086 (13)	0.0296 (15)
C4	0.0391 (18)	0.046 (2)	0.0565 (19)	0.0019 (14)	−0.0081 (16)	−0.0041 (18)
C5	0.062 (2)	0.045 (2)	0.064 (2)	0.0033 (19)	0.0009 (19)	−0.0012 (18)
C6	0.081 (3)	0.043 (2)	0.074 (2)	−0.0001 (19)	0.002 (2)	−0.014 (2)
C7	0.064 (2)	0.070 (2)	0.057 (2)	0.001 (2)	−0.0037 (17)	−0.020 (2)
C8	0.062 (2)	0.0448 (19)	0.0530 (19)	0.0070 (18)	−0.0061 (17)	0.0012 (16)
C9	0.0518 (18)	0.0384 (17)	0.0461 (16)	−0.0025 (15)	0.0024 (16)	−0.0004 (14)
C10	0.058 (2)	0.059 (2)	0.0439 (19)	0.0086 (18)	−0.0046 (18)	−0.0023 (15)
C11	0.070 (2)	0.061 (2)	0.0485 (19)	−0.002 (2)	0.0065 (19)	0.0034 (17)
C12	0.053 (2)	0.075 (3)	0.072 (3)	0.003 (2)	0.015 (2)	−0.002 (2)
C13	0.048 (2)	0.079 (3)	0.083 (3)	0.011 (2)	0.001 (2)	−0.006 (2)
C14	0.057 (2)	0.0475 (18)	0.0423 (18)	−0.0035 (17)	−0.0138 (16)	0.0029 (15)

### Geometric parameters (Å, °)

**Table:**

O1—C2	1.369 (3)	C5—H5A	0.9300
O1—C1	1.425 (4)	C6—C7	1.360 (5)
C1—H1A	0.9600	C6—H6A	0.9300
C1—H1B	0.9600	C7—H7A	0.9300
C1—H1C	0.9600	C8—C13	1.362 (4)
O2—C8	1.384 (3)	C8—C9	1.377 (4)
O2—C4	1.389 (3)	C9—C10	1.407 (4)
C2—C3	1.362 (4)	C9—C14	1.477 (4)
C2—C7	1.388 (4)	C10—C11	1.366 (4)
O3—C14	1.229 (3)	C10—H10A	0.9300
C3—C4	1.389 (4)	C11—C12	1.366 (4)
C3—H3A	0.9300	C11—H11A	0.9300
O4—C14	1.296 (3)	C12—C13	1.386 (5)
O4—H4A	0.8200	C12—H12A	0.9300
C4—C5	1.362 (4)	C13—H13A	0.9300
C5—C6	1.379 (4)		
			
C2—O1—C1	117.7 (2)	C6—C7—C2	119.7 (3)
O1—C1—H1A	109.5	C6—C7—H7A	120.1
O1—C1—H1B	109.5	C2—C7—H7A	120.1
H1A—C1—H1B	109.5	C13—C8—C9	121.8 (3)
O1—C1—H1C	109.5	C13—C8—O2	119.6 (3)
H1A—C1—H1C	109.5	C9—C8—O2	118.1 (3)
H1B—C1—H1C	109.5	C8—C9—C10	117.7 (3)
C8—O2—C4	120.0 (2)	C8—C9—C14	124.2 (3)
O1—C2—C3	124.2 (3)	C10—C9—C14	118.1 (3)
O1—C2—C7	116.2 (3)	C11—C10—C9	120.5 (3)
C3—C2—C7	119.6 (3)	C11—C10—H10A	119.8
C2—C3—C4	119.5 (3)	C9—C10—H10A	119.8
C2—C3—H3A	120.2	C10—C11—C12	120.6 (3)
C4—C3—H3A	120.2	C10—C11—H11A	119.7
C14—O4—H4A	109.5	C12—C11—H11A	119.7
C5—C4—O2	125.0 (3)	C11—C12—C13	119.8 (3)
C5—C4—C3	121.5 (3)	C11—C12—H12A	120.1
O2—C4—C3	113.4 (3)	C13—C12—H12A	120.1
C4—C5—C6	117.9 (3)	C8—C13—C12	119.7 (4)
C4—C5—H5A	121.1	C8—C13—H13A	120.2
C6—C5—H5A	121.1	C12—C13—H13A	120.2
C7—C6—C5	121.7 (3)	O3—C14—O4	122.0 (3)
C7—C6—H6A	119.1	O3—C14—C9	123.2 (3)
C5—C6—H6A	119.1	O4—C14—C9	114.8 (3)
			
C1—O1—C2—C3	3.1 (5)	C13—C8—C9—C10	−0.1 (5)
C1—O1—C2—C7	−177.4 (3)	O2—C8—C9—C10	171.1 (3)
O1—C2—C3—C4	179.6 (3)	C13—C8—C9—C14	178.6 (3)
C7—C2—C3—C4	0.1 (5)	O2—C8—C9—C14	−10.2 (5)
C8—O2—C4—C5	−9.0 (5)	C8—C9—C10—C11	1.3 (4)
C8—O2—C4—C3	171.1 (3)	C14—C9—C10—C11	−177.5 (3)
C2—C3—C4—C5	−1.3 (5)	C9—C10—C11—C12	−1.0 (5)
C2—C3—C4—O2	178.6 (3)	C10—C11—C12—C13	−0.6 (6)
O2—C4—C5—C6	−178.6 (3)	C9—C8—C13—C12	−1.5 (5)
C3—C4—C5—C6	1.3 (5)	O2—C8—C13—C12	−172.5 (3)
C4—C5—C6—C7	−0.1 (5)	C11—C12—C13—C8	1.8 (6)
C5—C6—C7—C2	−1.1 (6)	C8—C9—C14—O3	−16.7 (5)
O1—C2—C7—C6	−178.4 (3)	C10—C9—C14—O3	162.0 (3)
C3—C2—C7—C6	1.1 (5)	C8—C9—C14—O4	163.9 (3)
C4—O2—C8—C13	−67.7 (4)	C10—C9—C14—O4	−17.4 (4)
C4—O2—C8—C9	121.0 (3)		

### Hydrogen-bond geometry (Å, °)

**Table:**

Cg1 is the centroid of the C2–C7 ring.

**Table:**

*D*—H···*A*	*D*—H	H···*A*	*D*···*A*	*D*—H···*A*
O4—H4A···O3^i^	0.82	1.82	2.633 (3)	173
C1—H1B···Cg1^ii^	0.96	2.89	3.784 (4)	155

Symmetry codes: (i) −*x*+2, −*y*, −*z*+2; (ii) −*x*+3/2, *y*+1/2, *z*.
